# A policy brief on improving reproductive and maternity services utilisation among women of reproductive age in Nigeria

**DOI:** 10.3389/fgwh.2025.1608774

**Published:** 2025-09-05

**Authors:** Obasanjo Bolarinwa, Rebecca Tadokera, Ritika Tiwari

**Affiliations:** 1 Department of Business, Management and Health, York St. John University, London, United Kingdom; 2 Department of Demography and Population Studies, University of Witwatersrand, Johannesburg, South Africa; 3 Department of Health Sciences, York St. John University, York, United Kingdom

**Keywords:** policy brief, reproductive service, maternity service, women, Nigeria

## Abstract

Nigeria continues to struggle with high maternal and child mortality despite its large economy. Reproductive and maternity services are underutilised, contributing to poor maternal and newborn outcomes. Barriers include geographic, socio-demographic, and economic factors. Northern and south-south regions show particularly low service utilisation. Young maternal age, low education, rural location, and Hausa ethnicity are key socio-demographic barriers. Non-Christian religious affiliation and limited mass media exposure also reduce service uptake. Poverty, unemployment, and lack of health insurance are major economic challenges. Community engagement and culturally sensitive care are essential. The use of religious and traditional leaders for advocacy could improve outreach. Expanding insurance and financial incentives, like vouchers or cash transfers, can reduce cost-related barriers

## Highlights

- Nigeria continues to struggle with high maternal and child mortality despite its large economy.
- Reproductive and maternity services are underutilised, contributing to poor maternal and newborn outcomes.
- Barriers include geographic, socio-demographic, and economic factors
- Northern and south-south regions show particularly low service utilisation
- Young maternal age, low education, rural location, and Hausa ethnicity are key socio-demographic barriers
- Non-Christian religious affiliation and limited mass media exposure also reduce service uptake
- Poverty, unemployment, and lack of health insurance are major economic challenges
- Community engagement and culturally sensitive care are essential
- The use of religious and traditional leaders for advocacy could improve outreach
- Expanding insurance and financial incentives, like vouchers or cash transfers, can reduce cost-related barriers

## 1 Introduction

Reproductive and maternity services utilisation is important in preventing poor maternal and child outcomes, including maternal and newborn mortalities. Despite having one of the largest economies in Africa and a vast amount of human and material resources, Nigeria remains one of the few countries with an extremely high burden of maternal and newborn deaths in the world. In 2020, for instance, Nigeria was the only lower-middle-income country among the top three countries with extremely high maternal mortality rates in the world, recording 1,047 deaths per 100,000 live births, with the other two countries being Chad and South Sudan, both low-income countries (1). Besides, Nigeria alone accounted for 12% of the global share of maternal deaths, stillbirths, and neonatal deaths in 2020, recording 82,000 maternal deaths, 181,000 stillbirths, and 277,000 neonatal deaths (2). Meanwhile, most of these maternal and newborn deaths are preventable with adequate access to and utilisation of reproductive and maternity services, including antenatal care (ANC), skilled deliveries, and the use of modern contraceptives.

Women in Nigeria continue to face several barriers to accessing and utilising reproductive and maternity services. These include socio-demographic barriers such as lower educational attainment and younger maternal age (3), geographic factors such as long distance from healthcare facilities and rural residency, and economic barriers like poverty (4). Cultural and traditional beliefs also influence reproductive and maternal healthcare practices and health-seeking behaviour, promoting the use of traditional care during pregnancy and childbirth, including traditional birth deliveries (5). Besides, systemic barriers such as limited availability of services in some healthcare facilities, poor quality of care, stigma, and discriminatory and abusive practices of some healthcare workers also curtail the utilisation of reproductive and maternity services in Nigeria (4, 6).

Addressing the barriers to reproductive and maternity services utilisation is important not only in enhancing maternal and child health outcomes (7, 8) but also in ensuring economic, social, and community development, as well as gender empowerment and equality (9). A number of policies have been implemented to address the limited utilisation of reproductive and maternity services in Nigeria, including the Free Maternal and Child Health Services (not available in all states) (10), the Mothercare Nigeria Project (11), Integrated Maternal, Neonatal and Child Health (12), and the National Reproductive Health Policy and Strategy (13). Although these and similar policy interventions contributed to a decline in maternal morbidities and mortalities in Nigeria (10, 13, 14), the persistently high maternal and child morbidities and mortalities suggest policy deficiencies or gaps in addressing the problem (14). For instance, some of the policy interventions have been criticised for a lack of a targeted approach in addressing particular barriers hindering the use of reproductive and maternity services among specific segments of the population (13). Besides, limited resources, stakeholder engagements, and a lack of effective monitoring and coordination often hinder the implementation of policy interventions, contributing to policy failures (15). Although the recently launched multi-million dollar Nigeria Health Sector Renewable Investment Initiative, a partnership between the World Bank and the Nigerian government, aims to drastically reduce preventable deaths through targeted investment in primary healthcare (16), the scope of the initiative is too broad to address some of the specific barriers associated with reproductive and maternity services utilisation in Nigeria.

The recent maternal and newborn mortality estimates of Nigeria highlight the urgent need for targeted interventions to address the issues of reproductive and maternal healthcare in the country. Therefore, in this policy brief, we present a framework for improving reproductive and maternity services using evidence-based strategies.

## 2 Rationale and aim of the policy brief

This policy brief aims to advocate for measures to improve reproductive and maternity services utilisation in Nigeria. This will be realised by analysing the main barriers associated with low utilisation of reproductive and maternity services and recommending evidence-based policy interventions to effectively address the identified barriers. We envisage that the successful implementation of these recommendations will significantly improve reproductive and maternity services utilisation in Nigeria and enhance maternal and child well-being in the country.

The utilisation of reproductive and maternity services among women in Nigeria is a critical issue that needs urgent attention. For instance, an analysis of the 2018 Nigeria Demographic and Health Survey (NDHS) revealed that less than half (41%) of pregnant women in Nigeria were delivered by skilled birth attendants (17), and only 12% of women of reproductive age use modern contraceptives (18), emphasising the urgency of interventions to promote utilisation of reproductive and maternity services in the country. Whilst recent efforts and interventions to promote access and utilisation of reproductive and maternity services yielded some positive results (10, 12, 13), the persistently low utilisation levels suggest existing policy gaps and calls for renewed efforts and strategies to address the problem. For instance, the National Reproductive Health Policy of 2010 did not achieve its intended objectives due to its inability to effectively reach hard-to-reach communities and address population-specific needs (19). Addressing these gaps is essential in enhancing reproductive and maternal health outcomes among women, minimising the health, social and economic burden associated with poor maternal health.

## 3 Recommendations for policy reforms

In this section, evidence-based recommendations targeted at enhancing reproductive and maternity services utilisation through policy reforms are presented. The recommendations rely on current research findings and available best practices from various reputable sources, including the WHO, to provide effective and actionable interventions for implementation.

### 3.1 Providing geographically tailored interventions

Evidence shows wide geographic disparities in the utilisation of reproductive and maternity services, including ANC (20), skilled deliveries (17), and modern contraceptives (21), with a higher burden of non-use observed in the northern (22) and south-south regions (23) of Nigeria. This pattern of non-use has been attributed to factors such as cultural norms and geographic disparities (22, 23). Policymakers need to implement comprehensive measures targeted at these areas to promote the use of reproductive and maternal services in the country. Besides limitations in the physical distribution of healthcare facilities, evidence suggests that differences in cultural and religious beliefs also contribute to the geographical disparities in the utilisation of reproductive and maternity services in Nigeria (17, 20, 21). Therefore, policy interventions should focus on community engagement and awareness creation, enhanced advocacy through local leaders and community influencers such as religious leaders, chiefs, and traditional birth attendants (15). Additionally, building the capacity of healthcare workers, especially in providing culturally and religious sensitive reproductive and maternity services, could also enhance utilisation (24, 25).

### 3.2 Addressing socio-demographic barriers

The role of socio-demographic factors in hindering the utilisation of reproductive and maternity services (17, 20, 21) emphasises the need for interventions to mitigate the socio-demographic challenges and improve the utilisation of services. Policies should be targeted at addressing key socio-demographic barriers to promote access and utilisation. For instance, the WHO recommends the use of mobile health clinics to provide healthcare to individuals in hard-to-reach areas or those affected by a crisis (26). Thus, the deployment of mobile clinics to provide reproductive and maternity services in rural areas could enhance utilisation. Also, enhancing health literacy programmes and peer education using local language could promote the use of reproductive and maternity services among women with lower or no education (27).

### 3.3 Eliminating economic barriers and providing incentives

Studies show that economic factors such as poverty and unemployment are major contributors to the low utilisation of reproductive and maternity services in Nigeria (17, 20, 21). Policies aimed at eliminating economic barriers to healthcare access and utilisation need to be enhanced. For example, whilst evidence shows that health insurance coverage enhances women's utilisation of reproductive and maternity services in Nigeria (28), out-of-pocket payments remain a major policy gap in the implementation of health insurance packages in Nigeria and many other countries in sub-Saharan Africa (29, 30). Aside from curtailing healthcare utilisation and universal health coverage, out-of-pocket payments also push millions of Africans into poverty (31), increasing their inability to access and use healthcare services. Thus, policymakers should strengthen the monitoring and enforcement of health insurance policies, enhance public education and awareness, and ensure inclusive benefits of insurance policies for reproductive and maternity services to eliminate out-of-pocket payments (32). Also, ensuring that the free maternal healthcare policy is implemented in every state in Nigeria could enhance utilisation and improve maternal health outcomes (33). Although the World Bank recommends the provision of financial incentives through vouchers and conditional cash transfer systems to enhance the utilisation of reproductive and maternity services, particularly among poor women, it argues that increasing the income of women through employment and other income-generating activities including women-led small, medium-scale enterprises, and vocational training for girls and women could better enhances utilisation and health outcomes (34).

### 3.4 Enhancing the use of digital healthcare

Research has shown that utilisation of reproductive and maternity services in Nigeria is often limited by physical accessibility barriers due to geographic, socio-demographic, and economic difficulties (17, 20, 21). Policymakers should advocate and promote the use of digital healthcare platforms, including artificial intelligence (AI), among women of reproductive health age to circumvent some of the physical accessibility barriers and enhance utilisation and improve the health system in the country (35). In Rwanda, for instance, the integration of AI into its digital healthcare system has significantly enhanced the utilisation of healthcare services (36). Similar initiatives in Nigeria, targeted at reproductive and maternal healthcare, could significantly enhance utilisation in the country, reducing poor maternal and child health outcomes.

## 4 Conclusion

Ensuring adequate utilisation of reproductive and maternity services is essential in promoting the health and well-being of women and children and significantly contributes towards reducing maternal and newborn morbidities and mortalities in Nigeria. Addressing the identified reproductive and maternity services utilisation barriers and implementing new measures or enhancing existing measures could significantly contribute towards enhanced utilisation of reproductive and maternity services in Nigeria and thereby improve maternal and child health outcomes.

To adequately address the barriers to utilisation of reproductive and maternity services in Nigeria, this policy brief concluded that envisaged interventions should be targeted at addressing geographical, socio-demographic, and economic challenges associated with utilisation. Interventions focusing on community engagement and awareness creation, increased advocacy through local leaders and community influencers such as religious leaders, chiefs, and traditional birth attendants, and building the capacity of healthcare workers, especially in providing culturally and religious sensitive reproductive and maternity care, could enhance utilisation of services. Also, the use of mobile health clinics and health literacy programmes (using local language) could enhance reproductive and maternity services utilisation among women in rural areas and those with limited education, respectively. Addressing economic barriers by improving health insurance coverage, preventing out-of-pocket payments, and providing financial incentives through vouchers and conditional cash transfers could enhance utilisation.

## Appendix

The supplementary materials for this article can be found online at:

https://journals.plos.org/plosone/article?id=10.1371/journal.pone.0258844

https://journals.plos.org/plosone/article?id=10.1371/journal.pone.0259250

https://www.mdpi.com/2227-9032/9/10/1389

https://link.springer.com/article/10.1186/s40834-022-00187-8

https://link.springer.com/article/10.1186/s12905-024-03167-z
